# Operative treatment of pulmonary primitive neuroectodermal tumor: a case report and literature review

**DOI:** 10.1186/s13019-024-02563-8

**Published:** 2024-03-05

**Authors:** Yiyuan Zhang, Ke Shang, Jialin Li, Mengyao Sun, Xiaoying Gu

**Affiliations:** 1 Department of Thoracic surgery, The First Hospital of Jilin University, Changchun, 130021 China; 2 Department of Anesthesiology, The First Hospital of Jilin University, Changchun, 130021 China; 3 Department of Cardial Surgery, The First Hospital of Jilin University, Changchun, 130021 China

**Keywords:** Pulmonary primitive neuroectodermal tumors, Surgery with cardiopulmonary bypass, Clinical outcomes, Prognosis

## Abstract

**Background:**

Pulmonary primitive neuroectodermal tumor (PNET), a member of the Ewing sarcoma family of tumors, is a rare malignancy that is associated with a grim prognosis. To date, fewer than 30 cases of pulmonary PNET have been reported. In this case report, we present the clinical details of a 12-year-old girl with pulmonary PNET who underwent surgical treatment. We also conducted an analysis and summary of other relevant studies and the surgical outcomes.

**Case presentation:**

In May 2018, a 12-year-old girl was admitted with symptoms of cough and blood-tinged phlegm. A computed tomography scan revealed a large mass, measuring 12.9 cm × 8.1 cm, in the right middle and lower lungs. A percutaneous lung biopsy confirmed poorly differentiated tumor cells with a nested growth pattern. Immunohistochemical staining demonstrated positive expression of CD99, CD56, Vimentin, and Synaptophysin. The patient was diagnosed with pulmonary PNET. Following three cycles of neoadjuvant chemotherapy, a substantial reduction in tumor volume was observed. Subsequently, the patient underwent a surgical procedure involving pneumonectomy and partial resection of the left atrium with the assistance of cardiopulmonary bypass. The patient was discharged 37 days after surgery. During a three-year follow-up period, she exhibited no signs of tumor recurrence and has successfully returned to school.

**Conclusions:**

This case highlights the successful management of an advanced PNET with neoadjuvant chemotherapy, pneumonectomy, and partial resection of the left atrium employing cardiopulmonary bypass. The patient remained disease-free after three years. Our analysis of surgically treated cases indicates that neoadjuvant chemotherapy can contribute to improved prognoses for PNET patients. It is crucial to emphasize that complete surgical excision remains the cornerstone of treatment, underscoring the importance of surgeons considering radical surgical approaches whenever feasible for patients with pulmonary PNETs.

## Background

Primitive neuroectodermal tumor (PNET), a member of the Ewing sarcoma family of tumors (ESFT) [1], has a dismal prognosis [2]. Peripheral PNET commonly occurs in the chest wall, pelvis, paraspinal, retroperitoneum, limbs, abdomen and neck in children and adolescents [2]. Pulmonary PNET is rare, and fewer than 30 pulmonary PNET cases have been reported thus far [3].

In this study, we report a case of a 12-year-old girl with pulmonary PNET who underwent surgery involving cardiopulmonary bypass, marking the first such reported case. Additionally, we have conducted an analysis and summary of other relevant studies and the surgical outcomes.

## Case presentation

In May 2018, a 12-year-old girl was admitted to our facility with a two-month history of cough, accompanied by one month of coughing up blood-tinged phlegm and noticeable weight loss. Physical examination revealed solid percussion sounds in the lower right lung and reduced breath sounds. Laboratory investigations showed elevated levels of neuron-specific enolase (NSE) at 32.11 ng/ml, cancer antigen 125 (CA-125) at 121.6 U/mL, and carcinoembryonic antigen (CEA) at 4.97 ng/mL, suggesting the possibility of a tumor.

**Fig. 1:**
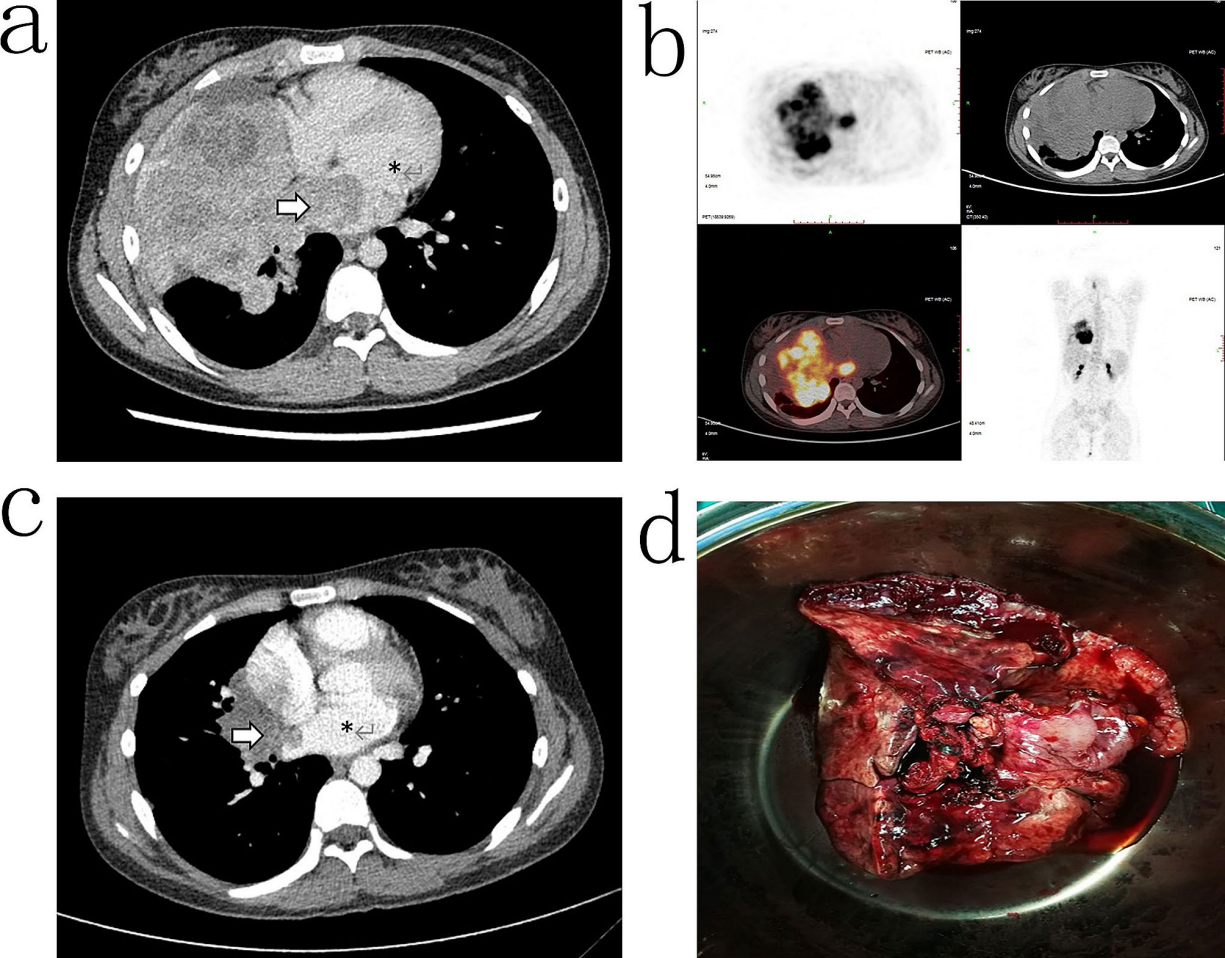
Tumor imaging of the patient diagnosed with pulmonary primitive neuroectodermal tumor. **a**: CT image at the first visit, **b**: PET result at the first visit, **c**: CT image after neoadjuvant chemotherapy, **d**: surgical specimens. Arrow: Tumor, *: Left atrium

Enhanced computed tomography (CT) scans revealed a large mass in the right middle and lower lung measuring 12.9 cm × 8.1 cm and displaying non-uniform enhancement (Fig. 1a). The tumor had infiltrated into the left atrium, compressed adjacent blood vessels, and occluded the middle bronchus of the right lung, accompanied by pleural effusion. Echocardiography detected a hyper-echoic region in the lateral thoracic cavity of the heart that had penetrated the pericardium and the left atrium wall, forming a space-occupying lesion in the left atrium.

Positron emission tomography (PET) revealed a large mediastinal mass with malignant potential and a maximum standardized uptake value (SUVmax) of 11.3 (Fig. 1b). No abnormalities were detected in other parts of the body. Bronchoscopy indicated complete blockage of the right middle bronchus by the tumor. Due to the risk of bleeding, biopsy through bronchoscopy was not feasible.

**Fig. 2:**
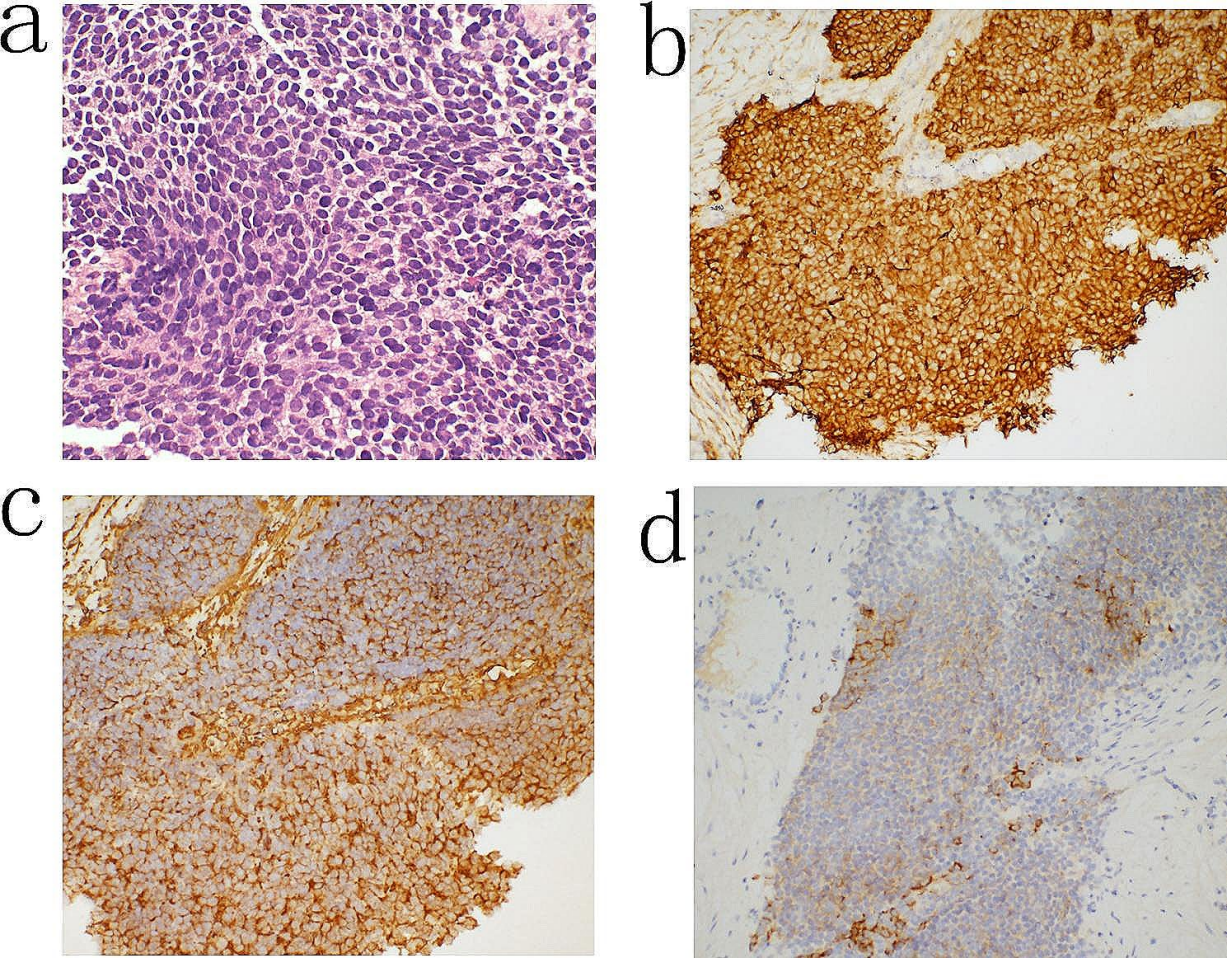
Pathological features of the pulmonary primitive neuroectodermal tumor. **a**: HE staining, **b**: CD99 staining (positive), **c**: Vimentin staining (positive), **d**: Syn foci (positive)

Percutaneous lung biopsy was then performed and showed poorly differentiated tumor cells with nestling growth, characterized by hyperchromatic nuclei and high nuclear-to-cytoplasmic ratios (Fig. 2a). Immunohistochemical staining demonstrated positive expression of CD99 (Fig. 2b), CD56, Vimentin (Fig. 2c), Syn (Fig. 2d) and high levels of the Ki67 proliferation marker. Based on the cell morphology and immunohistochemical findings, the patient was diagnosed with pulmonary PNET. Following three cycles of neoadjuvant chemotherapy involving cyclophosphamide, doxorubicin, and vincristine, a significant reduction in tumor volume was observed (Fig. 1c). The surgical procedure was performed with the patient in a supine position and the back elevated. A midline thoracotomy was conducted to establish extracorporeal circulation. During the surgery, the tumor was found to have extended into the left atrium.

After inducing cardiac arrest, complete removal of the tumor tissue that had extended into the pericardium was performed, along with atrial reconstruction. The main right pulmonary artery within the pericardium was isolated and ligated, and the tumor-invaded right pericardium was excised. Following these steps, the cardiopulmonary bypass was discontinued. After confirming normal cardiac activity, the right pulmonary artery and vein and the right main bronchus were dissected and ligated. The entire right lung and associated mediastinal lymph nodes were excised (Fig. 1d). The surgical incision was closed after thorough chest irrigation and placement of a drainage tube.

During the recovery period, the patient showed a sudden drop in blood pressure and an increase in heart rate that was unresponsive to vasoactive medications. Consequently, a secondary thoracotomy was performed, revealing rightward torsion of the heart due to absence of the pericardium and the entire right lung. Cardiac repositioning and repair of the pericardial defect were successfully carried out. The patient received postoperative ventilator support and made a satisfactory recovery.

Pathological examination results indicated the absence of lymph node metastasis. The patient was discharged 37 days after the surgical procedure. Adjuvant chemotherapy was declined by the patient’s family members. Throughout the three-year follow-up period, no evidence of tumor recurrence was observed, and the patient resumed her regular school activities.

## Discussion and conclusions

PNET is a member of the ESFT, which includes Ewing sarcoma of bone, extra-skeletal Ewing sarcoma, and Askin tumor [1]. A study of 54 patients revealed that PNETs can develop at any age, although they are predominantly observed in children and adolescents [2]. Pulmonary PNETs most frequently originate in the chest wall and pelvis and are less commonly found in the paraspinal region, retroperitoneum, limbs, abdomen, and neck. The incidence of PNET is higher in males compared with females (57% vs. 43%, respectively). Only 29 cases of pulmonary PNET have been documented to date, comprising 17 male and 12 female patients. Remarkably, approximately 82.8% of these 29 patients received a diagnosis of PNET before the age of 40 [3].

The patient in the current case report initially exhibited symptoms of cough and blood-tinged phlegm. PNET typically manifests with symptoms such as fever, chest pain, and shortness of breath (summarized in Table 1). CT imaging commonly reveals a solid mass located in the lung or near the mediastinum, often displaying significant enhancement. CT scans of PNET may also depict a mass with consistent densities and well-defined margins, which can remain relatively unchanged over a two-year period [4]. Pathological findings typically reveal the presence of small round cells characterized by poor differentiation, hyperchromatic nuclei, high nuclear-to-cytoplasmic ratios, and the formation of Homer–Wright rosettes. Immunohistochemical analysis commonly shows positivity for CD99, Vimentin, NSE, and Syn [5]. EWS-FLI-1 caused by t(11;22)(q24;q12) translocation is detected in approximately 85% patients, while t(21;12)(22;12) and other translocations are detected in approximately 10–15% of patients [1]. In the current study, the patient’s diagnosis was established through pathological examination and immunohistochemical profiling. However, it is important to consider differential diagnoses with other small round cell tumors, which may include small cell carcinoma, malignant lymphoma, Langerhans cell histiocytosis, rhabdomyosarcoma, neuroblastoma, and synovial sarcoma [6].

**Table 1:** Summary of published cases of primary pulmonary PNETs

Year	Author	Age/Sex	Location	Size (cm)	Symptoms	Neoadjuvant Therapy	Operation	Adjuvant Therapy	Follow-up (month)	Alive/Dead
1998	Tsuji [6]	25/F	LLL	3.6	Cough, fever, back pain	no	Lobectomy	-	24	D
		15/M	LLL	8.0	Asymptomatic	no	Lobectomy	CT	24	A
2000	Imamura [13]	41/M	LUL	5.2	Dyspnea	no	Resection	CT	22	A
		30/F	RLL	5.0	Cough, bloody sputum	CT	Lobectomy + Resection of partial left atrium	CT	16	A
2001	Baumgartner [14]	26/F	L hilum	-	Shortness of breath, chest pain	RT	Pneumonectomy	CRT	8	D
2001	Kahn [15]	18/M	RML	4.0	Dyspnea, cough, hemoptysis	no	Lobectomy	-	24	D
2001	Mikami [16]	17/F	RLL	5.5	Cough, fever	no	Lobectomy	CRT	6	D
2007	Lee [17]	67/M	LLL	4.0	Asymptomatic	no	Lobectomy	CT	-	-
2009	Demir [11]	22/F	Lung	13.0	-	CT	Lobectomy	CRT	32	A
		22/M	Lung	5.0	-	no	Lobectomy	CRT	18	D
		28/F	Lung	11.0	-	no	Lobectomy	CRT	15	A
		47/M	Lung	5.0	-	CT	Lobectomy + Resection of thoracic wall	CT	34	A
2009	Gaude [10]	28/M	L hilum	-	Chest pain, dyspnea	no	Tumor debulking	CRT	4	D
2009	Verfaillie [18]	33/M	RLL	7.0	Cough, high fever, chest pain, dyspnea	no	Lobectomy	RT	22	D
2012	Weissferdt [5]	22/M	RUL	5.0	Chest pain, cough	no	Lobectomy	no	-	-
		27/M	LUL	4.0	Shortness of breath, chest pain	no	Lobectomy	CT	24	D
		29/F	LUL	5.0	Chest pain, dyspnea	no	Lobectomy	CT	36	D
		31/M	RLL	6.0	Cough, dyspnea	no	Lobectomy	CT	54	D
		29/M	RUL	6.0	Chest pain, cough	no	Lobectomy	CT	-	-
		56/F	RML	9.6	Cough, fatigue	CT	Pneumonectomy	CT	11	A
2013	Shi [4]	19/M	LLL	5.5	Asymptomatic	no	Lobectomy	no	48	A
2016	Narayan [19]	8/F	R lung	9.0	Fever	no	Resection	CT	60	A
2016	Zhang [8]	30/F	LLL	10.5	Shortness of breath, chest pain	CT	Argon-helium knife cryosurgery	CT + Sunitinib	14	D
2017	Başgöz [20]	58/M	RLL	7.7	Asymptomatic	CT	Resection	CT	-	A
2021	Wang [3]	41/F	LUL	1.9	Cough, fever	no	Wedge resection	no	18	A
2021	Current case	12/F	R hilum	12.9	Cough, hemoptysis	CT	Pneumonectomy + Resection of partial left atrium	no	36	A

M: male, F: female, A: alive, D: dead, LUL: left upper lobe, LLL: left lower lobe, RUL: right upper lobe, RML: right middle lobe, RLL: right lower lobe, L: left, R: right, CT: chemotherapy, RT: radiotherapy, CRT: chemoradiation, -: no record

Pulmonary PNET is a rare tumor, with no standard treatment. Previous studies revealed that patients with pulmonary PNET have a high risk of recurrence and metastasis [7], leading to a dismal prognosis [2]. Surgery is generally the recommended treatment option for patients with resectable pulmonary PNET. In cases where surgery is not a feasible option, a combination of chemotherapy and radiotherapy should be considered as an alternative treatment approach. In one reported case, targeted therapeutic drugs were used in the treatment of pulmonary PNET [8].

Patients who are deemed ineligible for surgery, because of tumor metastasis and other medical conditions, experience a notably short survival period. To investigate the impact of various surgical approaches and adjuvant treatment regimens on the prognosis of patients with pulmonary PNET, we conducted a comprehensive review of all reported cases of pulmonary PNET in PubMed since 1998 (Table 1). A total of 26 patients who underwent surgical intervention were included in our analysis. The age of the patients ranged from 8 to 67 years, with tumor sizes spanning from 1.9 to 12.9 cm. The follow-up duration ranged from 4 to 60 months. Surgical procedures included lobectomy, pneumonectomy, and wedge resection. The overall mortality rate was 42.3%, with 11.5% of patients succumbing within one year following surgery (Table 2). The primary cause of death was attributed to tumor recurrence. Individuals with smaller tumors appeared to have the potential for achieving long-term survival [3].

**Table 2:** The outcome of patients with primary pulmonary PNET according to treatment

Treatment strategy		Number	1st-year death	Death toll	1st-year mortality	Total mortality
Operation		26	3	11	11.5%	42.3%
Lobectomy		17	1	8	5.9%	47.1%
Pneumonectomy		3	1	1	33.3%	33.3%
Extended resection*		3	0	0	0.0%	0.0%
Neoadjuvant Chemotherapy		7	0	1	0.0%	14.3%
No Neoadjuvant Chemotherapy		19	3	10	15.8%	52.6%
Adjuvant Chemotherapy		19	3	8	15.0%	45.0%
No Adjuvant Chemotherapy		5	0	1	0.0%	20.0%

*: Includes tumor resection with partial left atrium or thoracic wall

Pneumonectomy should be considered as the primary choice for pulmonary PNET cases, especially when the tumor size exceeds 5 cm and involves the hilus pulmonis tissue at the initial presentation. While the short-term risk associated with pneumonectomy is higher than that of lobectomy, the long-term risk of mortality is lower (33.3% vs. 47.1%). Although the number of cases analyzed in this study was limited, these findings suggest that extended resection may enhance the prognosis of advanced pulmonary PNET patients, resulting in a 100% increase in survival within one year. The present case is an example where the patient survived through the three-year follow-up and successfully returned to regular activities.

Existing research indicates that cardiac involvement should not be considered a contraindication for surgery. Although the use of cardiopulmonary bypass may slightly increase the risk of blood metastases, the benefits of achieving complete resection far outweigh the potential risk [9]. Therefore, complete resection should be considered as the first choice [10]. It is important to emphasize the necessity of pericardial patch repair because simultaneous excision of the affected pericardial tissue and lung may lead to cardiac torsion or heart hernia. Neoadjuvant chemotherapy has proven to be effective in the case of pulmonary PNET, significantly reducing the risk of postoperative mortality (14.3% vs. 52.6%). These findings are consistent with those of other studies [2, 11]. The efficacy of adjuvant chemotherapy remains controversial, as it does not result in a significant reduction in the risk of death [11, 12]. Patients who cannot achieve complete cure through surgery typically require adjuvant radiotherapy, and they often exhibit a less favorable prognosis. In the literature, patients who received postoperative adjuvant radiotherapy had a significantly higher 2-year mortality rate compared with those who did not (71.4% vs. 28.6%).

Pulmonary PNET is an uncommon malignancy with a poor prognosis that often originates from the chest wall and pelvis. Diagnosis of PNET typically involves various methods such as CT imaging, pathology, immunohistochemical analysis, and gene fusion detection. Here we presented a unique case involving an advanced PNET patient who underwent neoadjuvant chemotherapy, pneumonectomy, and partial resection of the left atrium with the assistance of cardiopulmonary bypass, marking the first such reported case. Remarkably, the patient remained disease-free for three years following treatment. Our analysis of surgically managed cases suggests that neoadjuvant chemotherapy can contribute to improved prognoses for PNET patients [11]. Complete surgical excision remains the cornerstone of treatment for pulmonary PNET. Therefore, it is advisable for surgeons to aim for radical surgical approaches whenever feasible. In cases where primary surgical resection is not possible, considering neoadjuvant chemotherapy as the initial treatment option is prudent. Subsequent evaluation for surgical resection should be performed based on the patient’s response to neoadjuvant therapy. The choice of postoperative chemotherapy should be made according to the patient’s condition. In situations where complete tumor removal is not achievable, a combination of radiotherapy or chemoradiation is recommended. Postoperative patients should undergo regular follow-up, including imaging examinations. Given the elevated risk of early recurrence and mortality associated with PNET, we recommend frequent reevaluations every three months during the first two years post-surgery. After this initial period, the follow-up interval can be extended.

## Data Availability

The datasets used and/or analyzed during the current study are available from the corresponding author on reasonable request.

## Abbreviations

CA-125: cancer antigen 125
CEA: carcinoembryonic antigen
CT: computed tomography
ESFT: Ewing sarcoma family of tumors
NSE: neuron-specific enolase
PET: positron emission tomography
PNET: primitive neuroectodermal tumor
